# Supplementary material for: Surpassing millisecond coherence in on chip superconducting quantum memories by optimizing materials and circuit design
Source: Nat Commun. 2024 May 1;15:3687. doi: 10.1038/s41467-024-47857-6 (PMC11063213; doi:10.1038/s41467-024-47857-6)
Supplement: Supplementary file 1 — Supplementary Information [file 41467_2024_47857_MOESM1_ESM.pdf]

Supplementary Information: Surpassing millisecond coherence in on chip superconducting quantum memories by optimizing materials and circuit design

Suhas Ganjam[1,2*], Yanhao Wang[1,2], Yao Lu[1,2], Archan Banerjee[1,2], Chan U Lei[1,2], Lev Krayzman[1,2], Kim Kisslinger[3], Chenyu Zhou[3], Ruoshui Li[3], Yichen Jia[3], Mingzhao Liu[3], Luigi Frunzio[1,2], Robert J. Schoelkopf[1,2*]

[1]Departments of Applied Physics and Physics, Yale University, New Haven, 06511, CT, USA.

[2]Yale Quantum Institute, Yale University, New Haven, 06511, CT, USA.

[3]Center for Functional Nanomaterials, Brookhaven National Laboratory, Upton, 11973, NY, USA.

*Corresponding author(s). E-mail(s): suhas.ganjam@yale.edu; robert.schoelkopf@yale.edu;

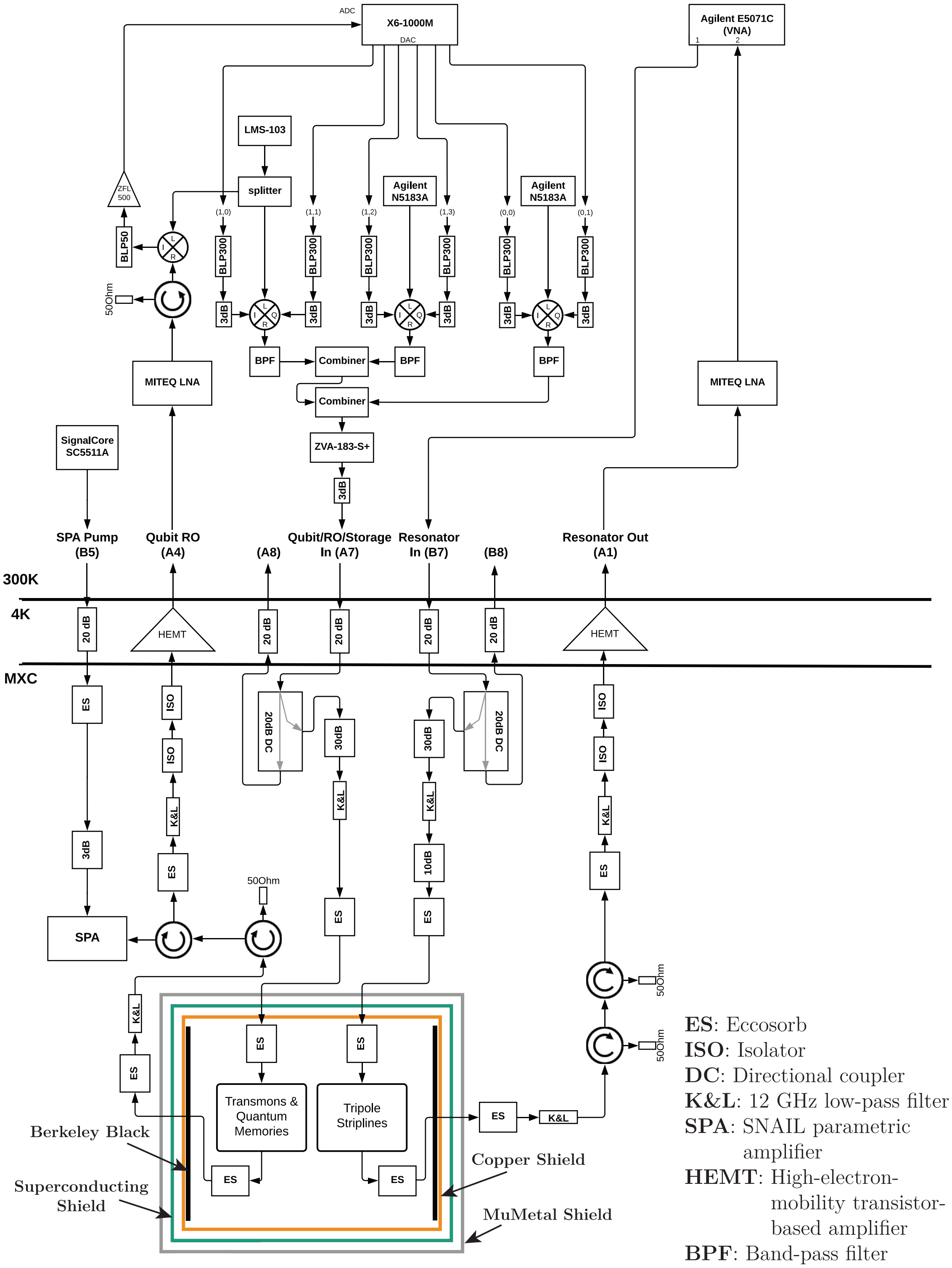

Fig. S1: Measurement setup

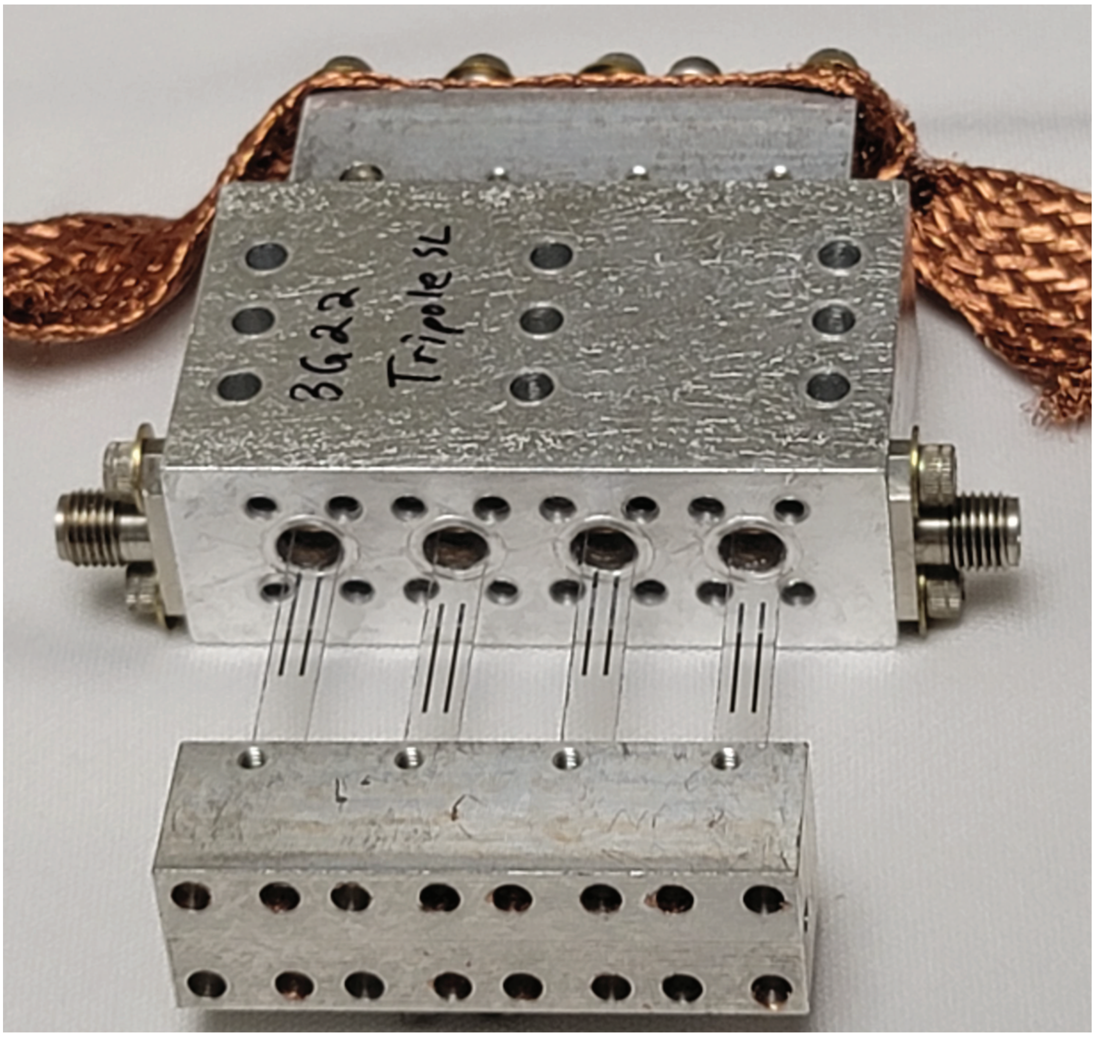

Fig. S2: **Device packaging**

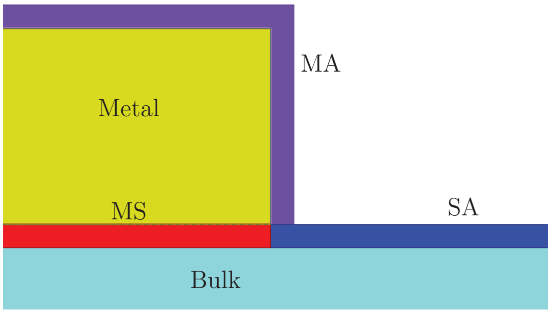

Fig. S3: **Surface dielectric interfaces**

Table S1: Summary of loss characterization devices

Device ID	Sapphire Growth Method	Anneal	Thin-Film Superconductor	Mode $Q_{\text{int}}(\overline{n}=1)$ $(\times 10^6)$ D1	D2	C	Loss Factors $(\overline{n}=1)$ Γ_{surf} $(\times 10^{-4})$	Γ_{bulk} $(\times 10^{-8})$	$1/g_{\text{seam}}$ (Ωm) $(\times 10^{-3})$
AM22 TSL1	HEM	No	Al	0.55	8.95	9.00	14.5 ± 1.0	7.96 ± 0.5	13.4 ± 0.9
AM22 TSL2				0.41	8.79	4.30	19.7 ± 3.2	5.65 ± 1.5	51.6 ± 2.4
AM22 TSL3				0.39	7.13	6.41	20.8 ± 6.3	8.78 ± 3.0	22.4 ± 1.2
DZ22 TSL3	HEM	Yes	Al	0.56	9.25	3.13	14.2 ± 3.5	7.42 ± 1.7	81.2 ± 1.2
DZ22 TSL4				0.82	5.41	6.81	8.58 ± 0.5	20.8 ± 0.4	13.1 ± 1.1
A23Al TSL2	HEMEX	Yes	Al	1.53	10.33	12.21	6.83 ± 0.4	4.14 ± 1.8	1.95 ± 1.6
A23Al TSL3				0.88	7.56	10.17	12.3 ± 0.5	2.57 ± 0.7	1.56 ± 0.3
EF21 TSL1	EFG	No	Ta	2.41	6.24	10.21	1.96 ± 0.2	19.8 ± 0.7	0.87 ± 0.7
EF21 TSL2				1.91	6.22	9.93	2.89 ± 0.1	19.5 ± 1.2	1.26 ± 1.2
EF21 TSL3				1.58	3.66	0.81	2.72 ± 0.2	33.7 ± 0.2	186 ± 1.0
EF21 TSL4				1.63	3.65	0.48	2.54 ± 0.3	33.4 ± 2.0	331 ± 1.5
EC21 ASL1	EFG	Yes	Ta	2.67[1]	-	5.04	1.76 ± 0.4	28.5 ± 0.7	-
EC21 ASL2				3.41[1]	-	10.99	3.42 ± 0.2	7.16 ± 0.7	-
EC21 ASL3				3.19[1]	-	10.16	4.40 ± 0.2	2.22 ± 1.0	-
EC21 ASL4				2.14[1]	-	15.38	6.77 ± 0.2	1.62 ± 0.8	-
R22 TSL1	HEM	Yes	Ta	1.59	24.04	24.20	5.00 ± 1.0	2.78 ± 0.5	1.08 ± 0.4
R22 TSL3				2.04	26.01	7.06	3.87 ± 0.2	2.83 ± 0.2	11.9 ± 1.4
R22 TSL4				1.78	19.66	13.69	4.35 ± 0.6	4.23 ± 0.3	3.92 ± 0.3
BF22 TSL1	HEMEX	Yes	Ta	1.24	19.57	14.25	6.44 ± 0.7	3.30 ± 0.5	2.38 ± 0.2
BF22 TSL2				2.53	27.40	14.96	3.07 ± 0.1	2.95 ± 0.1	2.81 ± 0.2
BF22 TSL3				2.38	41.23	10.80	3.40 ± 0.3	1.25 ± 0.2	5.18 ± 0.2
BF22 TSL4				2.44	29.47	16.54	3.22 ± 0.1	2.56 ± 0.2	2.45 ± 0.3

[1]ASL differential (D) mode Q

TSL: Tripole stripline
ASL: Adjacent stripline

Table S2: Summary of seam loss

Device ID	$1/g_{\text{seam}}$ (Ωm) ($\times 10^{-3}$)	Median Relative Deviation (MRD)
AM22 TSL1	13.4 ± 0.9	1.9
AM22 TSL2	51.6 ± 2.4	10.3
AM22 TSL3	22.4 ± 1.2	3.9
DZ22 TSL3	81.2 ± 1.2	16.8
DZ22 TSL4	13.1 ± 1.1	1.9
A23Al TSL2	1.95 ± 1.6	0.6
A23Al TSL3	1.56 ± 0.3	0.7
EF21 TSL1	0.87 ± 0.7	0.8
EF21 TSL2	1.26 ± 1.2	0.7
EF21 TSL3	186 ± 1.0	39.9
EF21 TSL4	331 ± 1.5	71.8
R22 TSL1	1.08 ± 0.4	0.8
R22 TSL3	11.9 ± 1.4	1.6
R22 TSL4	3.92 ± 0.3	0.1
BF22 TSL1	2.38 ± 0.2	0.5
BF22 TSL2	2.81 ± 0.2	0.4
BF22 TSL3	5.18 ± 0.2	0.1
BF22 TSL4	2.45 ± 0.3	0.5
Average (excl. MRD > 3)	4.75 ± 4.5	-

Note: MRD $= |X_i - \tilde{X}|/\tilde{X}$, where $\tilde{X}$ is the median. MRD > 3 is chosen such that outliers are $> 4\sigma$ away from the mean.

Table S3: Average surface & bulk loss factors

Material/Process System	Γ_{surf} ($\times 10^{-4}$)
Al (Unannealed)	18.3 ± 2.7
Al (Annealed)	10.5 ± 2.9
Ta (Unannealed)	2.53 ± 0.4
Ta (Annealed)	4.15 ± 1.4

Material/Process System	Γ_{bulk} ($\times 10^{-8}$)
EFG (Unannealed)	26.6 ± 6.9
EFG (Annealed)	3.64 ± 2.5
HEM (Unannealed)	7.46 ± 1.3
HEM (Annealed)	4.31 ± 1.9
HEMEX (Annealed)	2.80 ± 0.9

Note: Outliers: MRD > 3.

Supplementary Note 1: Design of loss characterization devices

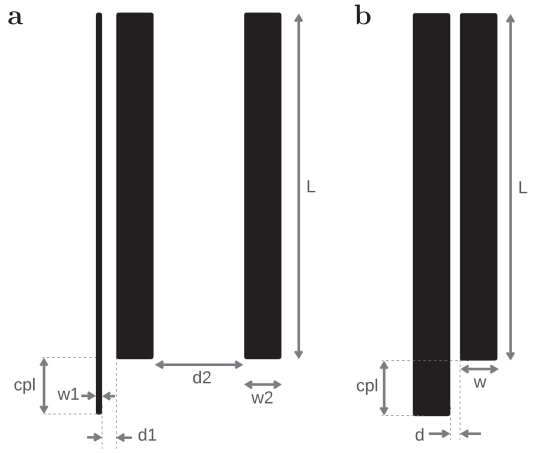

Fig. S4: **Multimode stripline device design. a** Tripole stripline (TSL). **b** Adjacent stripline (ASL).

Several different device designs were used in this work (Fig. S4). Tripole striplines (TSL) had 3 different designs that differed in stripline length (L), wide conductor width ($w2$), and narrow conductor spacing ($d1$). The dimensions of the TSL are given in Table S4. TSLv1 and TSLv2 are nearly identical in design except for the stripline length. TSLv2 is shorter to increase the mode frequency, allowing us to benefit from slightly higher gain from our HEMT amplifier at those frequencies. TSLv3, on the other hand, had different values of $d1$ and $w2$, in order to increase consistency and reproducibility during patterning with electron-beam lithography. In all TSL devices, the narrow stripline was longer than the wide striplines by length cpl; this introduces a field perturbation in the D1 mode that increases its coupling to the drive line, allowing the mode to be excited during measurement.

Devices EC21 ASL1-4 were measured using a different device called the adjacent stripline (ASL). Rather than having three striplines placed next to each other, the ASL contains two striplines that are spaced by a distance $d = 10$ μm apart. This device manifests two modes; a surface-sensitive differential (D) mode and a bulk and package-sensitive common (C) mode. While this device cannot distinguish between package seam loss and bulk dielectric loss, we measured these devices in the same tunnel package as another set of devices, BF22, from which we had extracted the device-specific seam losses. By using these extracted seam losses, we subtracted the seam loss contribution to the modes of the ASL and contracted the participation matrix to solve for the surface and bulk loss factors. The dimensions of the ASL are given in Table S4. It should be noted that the ASL was meandered in order to confine the eigenfield to slightly mitigate seam loss.

Participation matrices are given for the different types of tripole striplines and the adjacent stripline in Table S5. Because the substrate thickness differed for some devices, the participations slightly changed even among identical device designs.

The participation matrix of a loss characterization device determines its measurement sensitivity; that is, the lowest loss factor that can be resolved with a fractional error $\sigma_i/\Gamma_i < 1$[1]. This sensitivity is dependent on the loss factors themselves. We calculate the measurement sensitivity (Fig. S5) for TSLv1, TSLv3, and ASLv1 by fixing the package losses using previously measured package conductor and MA loss factors[1] and the average measured package g_{seam} excluding outliers (Fig. S2). We note that the sensitivities for TSLv1 and TSLv2 do not differ significantly, due to their participations being very similar to each other. From the sensitivity plots, we see that both the TSLv1 and TSLv3 designs can resolve bulk loss factors as low as 2×10^{-9} and surface loss factors as low as 1×10^{-6}. However, the inability

Table S4: Tripole & adjacent stripline dimensions

Dimension	Device Type TSLv1	TSLv2	TSLv3	ASLv1
cpl	0.5 mm	0.5 mm	0.5 mm	0.3 mm
L	14 mm	12 mm	12 mm	14 mm[1]
$w1$	10 µm	10 µm	10 µm	-
$w2$	400 µm	400 µm	100 µm	-
$d1$	10 µm	10 µm	20 µm	-
$d2$	1200 µm	1200 µm	1200 µm	-
d	-	-	-	10 µm
w	-	-	-	150 µm

[1]This stripline was meandered.

Table S5: TSL and ASL participation matrices

Device	Type	Substrate Thickness (µm)	Mode	Freq (GHz)	Participation Matrix p_{surf}	p_{bulk}	$p_{\mathrm{pkg}_{\mathrm{cond}}}$	$p_{\mathrm{pkg}_{\mathrm{MA}}}$	y_{seam} $(\Omega\mathrm{m})^{-1}$
AM22, DZ22	TSLv1	430	D1	4.52	1.2×10^{-3}	0.90	4.3×10^{-8}	5.9×10^{-10}	7.9×10^{-9}
			D2	5.34	3.5×10^{-5}	0.73	3.4×10^{-6}	2.9×10^{-8}	1.3×10^{-8}
			C	6.64	2.2×10^{-5}	0.38	1.2×10^{-5}	1.2×10^{-7}	3.1×10^{-6}
A23Al	TSLv3	650	D1	5.24	9.0×10^{-4}	0.90	1.5×10^{-7}	1.6×10^{-9}	8.6×10^{-6}
			D2	5.51	8.9×10^{-5}	0.85	1.7×10^{-6}	1.4×10^{-8}	3.4×10^{-8}
			C	6.68	5.2×10^{-5}	0.54	8.7×10^{-6}	9.7×10^{-8}	9.3×10^{-6}
EF21	TSLv1	530	D1	4.52	1.2×10^{-3}	0.90	4.9×10^{-8}	6.1×10^{-10}	1.5×10^{-8}
			D2	5.14	3.4×10^{-5}	0.76	3.6×10^{-6}	2.9×10^{-8}	2.0×10^{-8}
			C	6.45	2.1×10^{-5}	0.41	1.2×10^{-5}	1.2×10^{-7}	5.8×10^{-6}
EC21	ASLv1	530	D	3.68	6.7×10^{-4}	0.90	6.5×10^{-8}	4.8×10^{-10}	3.2×10^{-8}
			C	6.03	2.8×10^{-5}	0.55	4.4×10^{-6}	7.5×10^{-8}	1.3×10^{-5}
R22	TSLv1	650	D1	4.52	1.2×10^{-3}	0.90	6.0×10^{-8}	6.5×10^{-10}	2.5×10^{-8}
			D2	4.97	3.4×10^{-5}	0.79	3.9×10^{-6}	2.9×10^{-8}	2.6×10^{-8}
			C	6.27	2.1×10^{-5}	0.43	1.8×10^{-5}	1.3×10^{-7}	9.4×10^{-6}
BF22	TSLv2	650	D1	5.25	1.2×10^{-3}	0.90	8.1×10^{-8}	8.5×10^{-10}	4.0×10^{-8}
			D2	5.74	3.5×10^{-5}	0.80	3.8×10^{-6}	2.7×10^{-8}	3.6×10^{-8}
			C	7.13	2.2×10^{-5}	0.45	1.3×10^{-5}	1.3×10^{-7}	1.4×10^{-5}

for the ASL to distinguish between bulk and seam loss reduces its sensitivity by an order of magnitude. Regardless, these sensitivities are still below what we actually resolve in measured devices, indicating that these loss characterization devices are well conditioned to probe losses in our regime of interest.

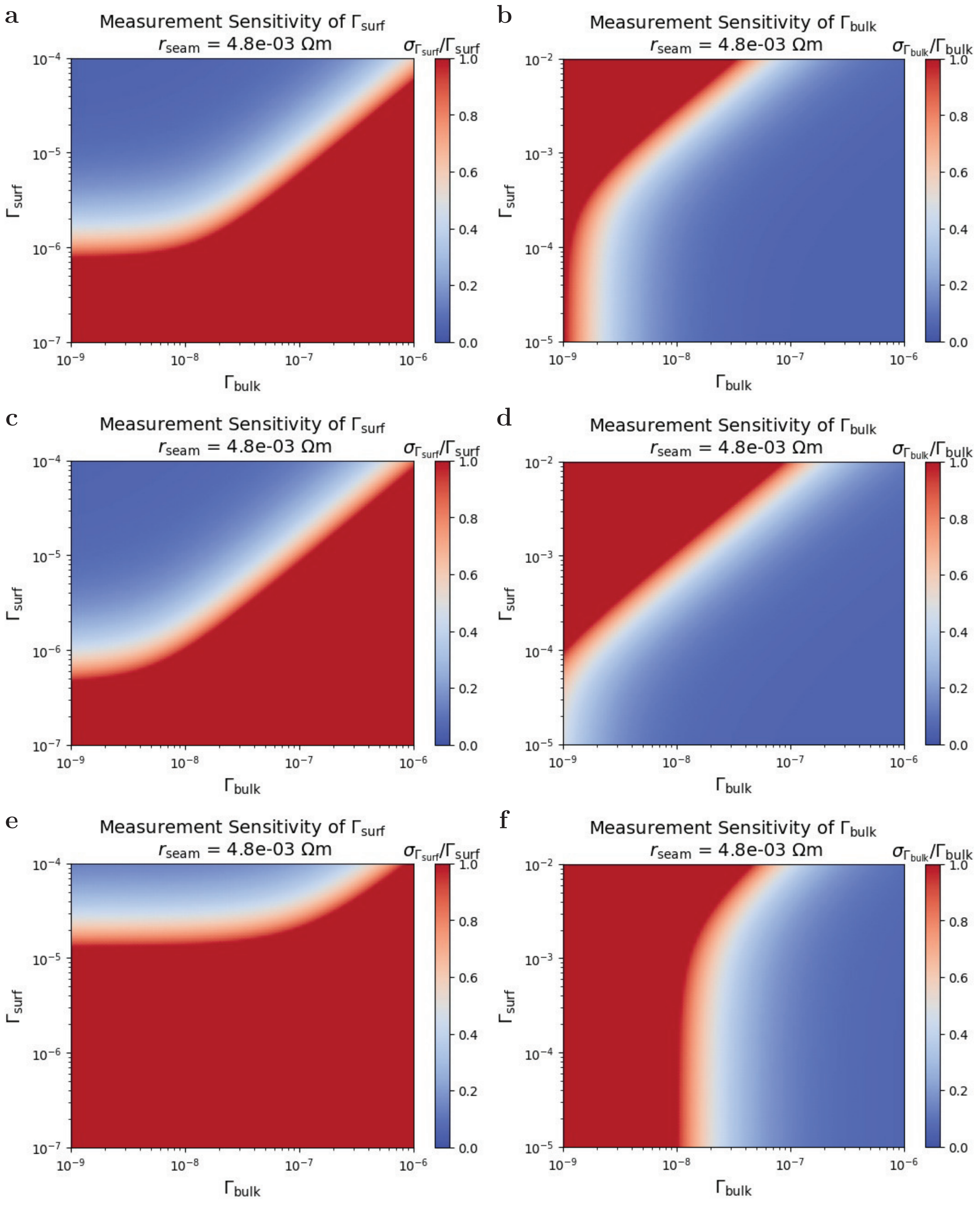

Fig. S5: **Surface and bulk loss measurement sensitivity** for TSLv1 (**a, b**), TSLv3 (**c, d**), and ASLv1 (**e,f**). Here, $r_{\text{seam}} = 1/g_{\text{seam}}$.

Supplementary Note 2: Extracting Ta/Al contact loss

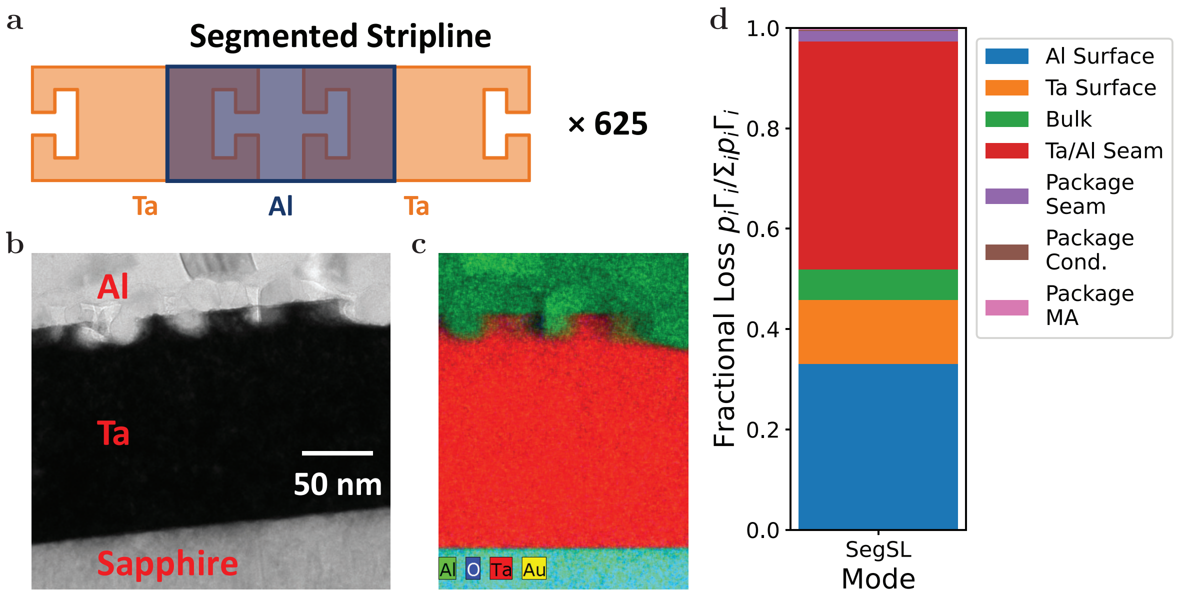

Fig. S6: **Segmented stripline to measure Ta/Al contact loss. a** Segmented stripline design; alternating segmented of Ta and Al are repeated to make the full length of the stripline. **b** TEM of Ta/Al interface, showing damage to the Ta film caused by the ion beam cleaning prior to Al deposition. **c** Elemental map formed by energy-dispersive X-ray spectroscopy of the TEM sample shows no oxide in the Ta/Al interface, indicating that good metal-to-metal contact is present. **d** Single-photon loss budget for a representative segmented stripline, showing that 50% of the device's total loss is due to Ta/Al contact loss.

Contact loss due to the interface between the Ta and Al films may contribute significantly to the total loss of a transmon qubit. Tantalum oxide or other contaminants located in the tantalum/aluminum interface may contribute to an effective resistance in series with the transmon's Josephson junction. To alleviate this loss, we employ an argon ion beam cleaning step prior to aluminum deposition to remove the tantalum oxide and any potential contamination. TEM of the resulting Ta/Al interface reveals no oxide between Al and Ta, showing that ion beam clean effectively removes the tantalum oxide (Fig. S6b, c). However, the ion beam also appears to have damaged and roughened the Ta film, possibly introducing lossy defects. To characterize and quantify this loss channel, we designed the segmented stripline, a thin-film resonator made up of around 625 alternating segments of Ta and Al that contact each other, resulting in a 10 μm wide, 12.5 mm long stripline that is over 100 times more sensitive to contact loss than a regular transmon (Fig. S6a)[2]. We model the participations of this device by simulating the surface, bulk, and package participations using an electromagnetic simulation with the same method as was used for the tripole striplines. We use a seam loss model to describe the Ta/Al contact loss[3] similar to the package seam loss, where the geometric component of the loss is described by a seam admittance per unit length, $y_{\mathrm{seam}_{\mathrm{Ta/Al}}}$, and an intrinsic loss factor described as the inverse of the seam conductance per unit length, $1/g_{\mathrm{seam}_{\mathrm{Ta/Al}}}$. Since the current mostly flows along the propagation axis of the stripline, we can assume a seam length equal to the stripline width $w = 10$ μm, and convert the seam conductance into a contact resistance, $R_{\mathrm{Ta/Al}} = (w \cdot g_{\mathrm{seam}_{\mathrm{Ta/Al}}})^{-1}$. To calculate $y_{\mathrm{seam}_{\mathrm{Ta/Al}}}$, we use an analytical model that assumes a sinusoidal current distribution throughout the stripline:

$$y_{\text{seam}_{\text{Ta/Al}}} = \frac{2}{\pi}\frac{\sum_i \sin^2(\pi z_i/l)}{wZ_0}, \quad (1)$$

where l is the total length of the stripline, z_i is the position along the propagation axis of the ith Ta/Al contact, w is the width of the stripline, and Z_0 is the characteristic impedance of the stripline mode. The participations of the segmented stripline are given in Table S6. The device was fabricated on annealed HEM sapphire using the same process as the tantalum-based transmon. Since we have already characterized the losses associated with the Al surface, Ta surface, bulk substrate, and package, we only need the quality factor measurement of the segmented stripline to extract the Ta/Al contact loss. Two nominally identical segmented striplines were measured at single photon powers. Their quality factors were very similar, differing by less than 10% (Table S7). Loss extraction for both devices revealed that around 50% of the total loss from the segmented stripline was due to the Ta/Al contact loss, which verifies that our loss characterization device is sensitive to the loss channel of interest (Fig. S6d). We therefore calculated an average seam resistance of 260 ± 47 nΩ.

Table S6: Segmented stripline participations

Freq (GHz)	5.74
$p_{\text{surf}_{\text{Ta}}}$	1.6×10^{-4}
$p_{\text{surf}_{\text{Al}}}$	1.6×10^{-4}
p_{bulk}	0.72
$y_{\text{seam}_{\text{Ta/Al}}}$	9.4×10^{4}
$p_{\text{pkg}_{\text{cond}}}$	3.3×10^{-6}
$p_{\text{pkg}_{\text{MA}}}$	4.8×10^{-8}
$y_{\text{pkg}_{\text{seam}}}$	2.3×10^{-6}

Table S7: Segmented stripline loss

Device	$Q_{\text{int}}(\overline{n} = 1)$	$R_{\text{Ta/Al}}$ (nΩ)
SegSL1	1.97×10^6	246 ± 59
SegSL2	1.88×10^6	272 ± 73

As with the segmented stripline, the Ta/Al contact participation of the tantalum-based transmon $y_{\text{seam}_{\text{Ta/Al}}}$ was calculated analytically. We assume a lumped-element model, where the contact resistance is modeled as a capacitor shunting a resistor in series with the junction. The quality factor due to the contact loss can then be straightforwardly written down as $Q_{\text{Ta/Al}} = Z_0/2R_{\text{Ta/Al}}$, where $Z_0 = \sqrt{L_J/C}$ is the characteristic impedance of the transmon mode and the factor of 2 in the denominator accounts for the presence of two Ta/Al contacts in the tantalum-based transmon. Therefore, the Ta/Al seam admittance becomes $y_{\text{seam}_{\text{Ta/Al}}} = 2/Z_0 w$, where $w = 10$ µm is the length of the seam. For our transmon design, this gives $y_{\text{seam}_{\text{Ta/Al}}} = 737.86$ $(\Omega\text{m})^{-1}$. Using the extracted value of $R_{\text{Ta/Al}} = 260 \pm 47$ nΩ from the segmented striplines, we estimate the loss due to Ta/Al contact resistance to limit the transmon Q_{int} to a maximum of approximately 5×10^8.

Supplementary Note 3: Transmon qubit device design

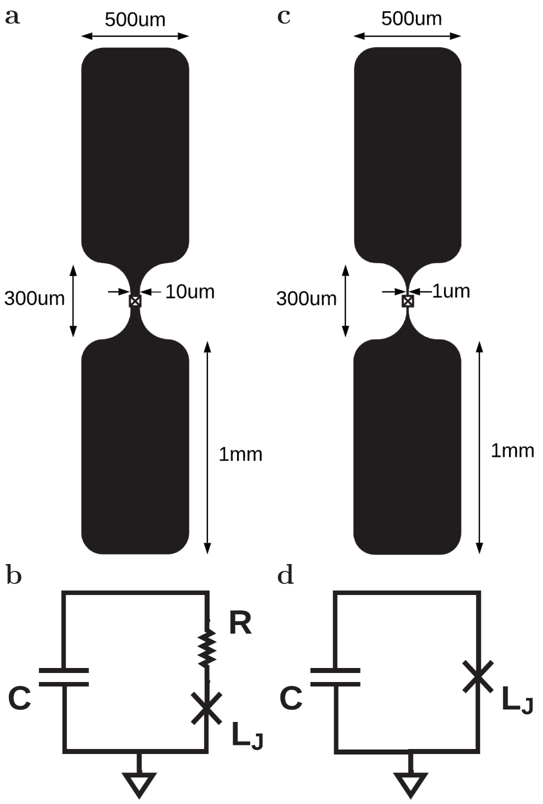

Fig. S7: **Transmon qubit device design. a, b** Ta-based transmon and circuit model. The resistor is used to represent Ta/Al contact loss. **c, d** Al-based transmon and circuit model. Junction leads are thinner to improve reliability and reproducibility of the electron-beam lithography process.

We utilized a standard 3D transmon design used for cavity-based cQED experiments[4] (Fig. S7). Each device contains a transmon coupled to a stripline readout resonator patterned on a chip that is inserted into a cylindrical tunnel. The chip also contains a bandpass Purcell filter to obtain a large external coupling of the drive line to the readout resonator while preserving a low external coupling of the drive line to the transmon (Table S9). Tantalum- and aluminum-based transmons had very similar designs, with some exceptions. In the tantalum-based transmon, the tantalum leads from the capacitor to the Josephson junction are wider at 10 μm (Fig. S7a). For the aluminum-based transmon, patterning such a large feature so close to the junction using only electron-beam lithography resulted in significant proximity dosing which severely impacted device yield. As a result, Al-based transmons were patterned with 1 μm-wide junction leads (Fig. S7b). This design change resulted in slightly higher overall surface participation for the aluminum-based transmon (Table S8).T

Table S8: Transmon participations

	Al transmon	Ta transmon
$p_{\mathrm{surf}_{\mathrm{Ta}}}$	-	8.1×10^{-5}
$p_{\mathrm{surf}_{\mathrm{Al}}}$	1.5×10^{-4}	5.5×10^{-5}
p_{bulk}	0.84	0.84
$y_{\mathrm{seam}_{\mathrm{Ta/Al}}}$	-	7.4×10^{2}
$p_{\mathrm{pkg}_{\mathrm{cond}}}$	9.3×10^{-8}	9.3×10^{-8}
$p_{\mathrm{pkg}_{\mathrm{MA}}}$	5.1×10^{-9}	5.1×10^{-9}
$y_{\mathrm{pkg}_{\mathrm{seam}}}$	3.0×10^{-9}	3.0×10^{-9}

Table S9: Typical transmon & readout parameters

$\omega_{\mathrm{t}}/2\pi$ (GHz)	4.5-5.1
$\omega_{\mathrm{r}}/2\pi$ (GHz)	9.0-9.3
$\chi_{\mathrm{tt}}/2\pi$ (MHz)	170-180
$\chi_{\mathrm{tr}}/2\pi$ (MHz)	0.5-1.1
$\kappa_{\mathrm{r}}/2\pi$ (MHz)	0.5-1.1

Supplementary Note 4: Hairpin stripline device design and measurement

The hairpin stripline (Fig. S8) is optimized to minimize package loss in order to maximize quantum memory coherence. The insensitivity to package loss is obtained by folding a half-wave (length $2L$) stripline resonator into itself, resulting in a fundamental mode that is in some sense "differential"; electric field lines do not terminate at the walls of the package. Additionally, the current antinode is positioned at a location where the current flow is perpendicular to the axis of the cylindrical tunnel, resulting in minimal induced current along the walls of the package. Maximizing the coherence of the hairpin stripline requires using the materials and processes that yield the lowest intrinsic loss as well as minimizing surface and bulk dielectric participation by optimizing design. The latter is accomplished by optimizing the width of the stripline w, the spacing between the arms of the hairpin s, and the tunnel radius. Increasing w slightly reduces surface participation, while increasing s reduces both bulk and surface participation. However, increasing either while keeping the tunnel radius fixed results in increasing the package participation. For the losses extracted in this work and a fixed tunnel radius of 2.5 mm, we found the optimal values for w and s to be 800 μm and 1200 μm, respectively (Table S10).

The ancilla transmon couples to both the memory mode and the higher order stripline mode that acts as a readout mode. The readout mode is equivalent to a full-wave resonance mode, where electric field antinodes are located at both ends of the hairpin. The opposite polarities at either end of the hairpin gives rise to an electric field pattern that is orthogonal to that of the memory mode. To couple to both modes simultaneously, we stagger the transmon's capacitor pads, giving it a net dipole moment that points diagonally with respect to the memory and readout mode's fields.

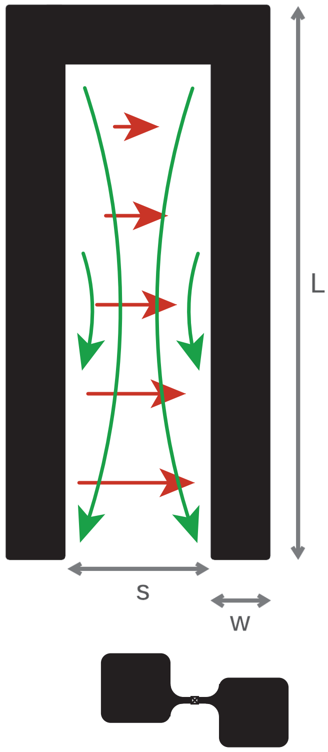

Fig. S8: **Hairpin stripline device design.** Field behaviors of the memory mode (red arrows) and readout mode (green arrows) are shown. The ancilla transmon has a staggered capacitor pad design that allows coupling to both modes.

Table S10: Hairpin stripline participations

p_{surf}	2.4×10^{-5}
p_{bulk}	0.72
$p_{\mathrm{pkg}_{\mathrm{cond}}}$	6.7×10^{-6}
$p_{\mathrm{pkg}_{\mathrm{MA}}}$	5.4×10^{-8}
y_{seam}	3.9×10^{-8}

Supplementary Note 5: Temporal fluctuations of coherence in transmons, quantum memories, and resonators

Transmon qubit coherence was measured over a period of at least 10 hours to capture temporal fluctuations (Fig. S10a), with some devices being measured over two days. We observed significant fluctuation in both T_1 and T_2 over long timescales, with T_1 fluctuating by around ±30% about the mean. In most devices, $T_{2,\mathrm{E}}$ was almost a factor of 2 higher than $T_{2,\mathrm{R}}$ but not as high as $2T_1$, indicating that low frequency and high frequency noise are present; we attribute the high frequency noise to thermal photon shot noise from the dispersively-coupled readout resonator[5]. We attribute temporal behavior of T_1 to fluctuating TLSs near and inside the Josephson junction, where the electric field densities due to the parasitic capacitance of the junction electrodes can by very high. We compare this behavior to that of the D1 mode Q over time of an aluminum tripole stripline (Fig. S10b). The internal Q of the D1 mode, despite

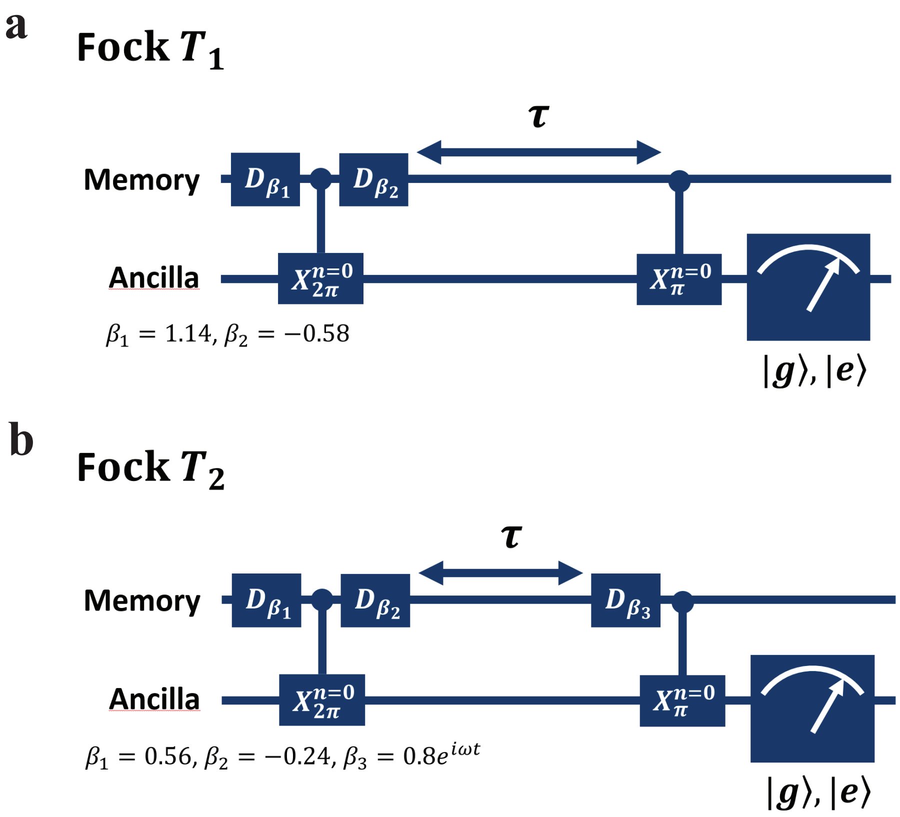

Fig. S9: **Quantum memory coherence measurement pulse sequences. a** The Fock $|1\rangle$ state was prepared using a pair of displacement pulses with a 2π rotation of the qubit in between. Measurement of the state is performed after a variable delay by performing a π pulse conditioned on the memory mode being in the $|1\rangle$ state. **b** A superposition of Fock $|0\rangle$ and $|1\rangle$ was prepared in a similar way but with different displacement amplitudes. After a variable delay, a third displacement (slightly off-resonant) is performed that interferes with the memory state and maps it back to either Fock $|0\rangle$ or $|1\rangle$, followed by measurement.

having higher surface participation than the transmon, fluctuates by only ± 10 % around its average value, due to the resonator's much larger area and more uniformly distributed electric field. Finally, we measure quantum memory coherence over time (Fig. S10c), and see that similar to the resonator and in sharp contrast with the transmon, the quantum memory T_1 and T_2 are quite stable over long timescales, with T_1 and T_1-limited T_2 fluctuating by only around 10% of their average values. From this, we can estimate that the majority of the transmon's T1 fluctuation comes from interactions with TLSs inside or within 100 nm from the junction, while around 10% of the fluctuations can be attributed to TLSs interacting with the rest of the transmon circuit, behavior that is already captured by resonator measurements.

Table S11: Typical quantum memory parameters

$\omega_\mathrm{m}/2\pi$ (GHz)	3.9-4.0
$\omega_\mathrm{t}/2\pi$ (GHz)	5.7-6.8
$\omega_\mathrm{r}/2\pi$ (GHz)	9.0-9.3
$\chi_\mathrm{tt}/2\pi$ (MHz)	201-217
$\chi_\mathrm{tm}/2\pi$ (MHz)	0.1-0.4
$\chi_\mathrm{tr}/2\pi$ (MHz)	0.3-0.6
$\kappa_\mathrm{r}/2\pi$ (MHz)	0.2-0.5

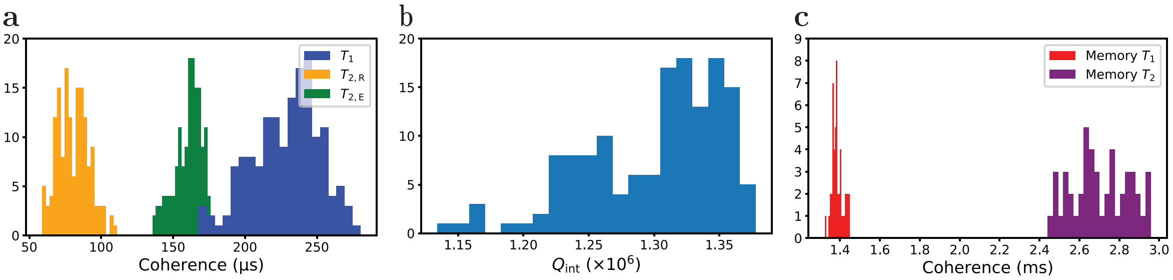

Fig. S10: **Temporal fluctuations in coherence. a** Representative histogram of temporal fluctuations for coherence in a Ta-based transmon device measured over 48 hours. **b** Representative histogram of temporal fluctuations for Q_int in a tripole stripline D1 mode measured over 35 hours. Data was taken at low power in the TLS-dominated regime, with $\overline{n} \sim 100$. **c** Representative histogram of temporal fluctuations in coherence in a hairpin stripline quantum memory device measured over 30 hours.

Supplementary Note 6: Sapphire annealing

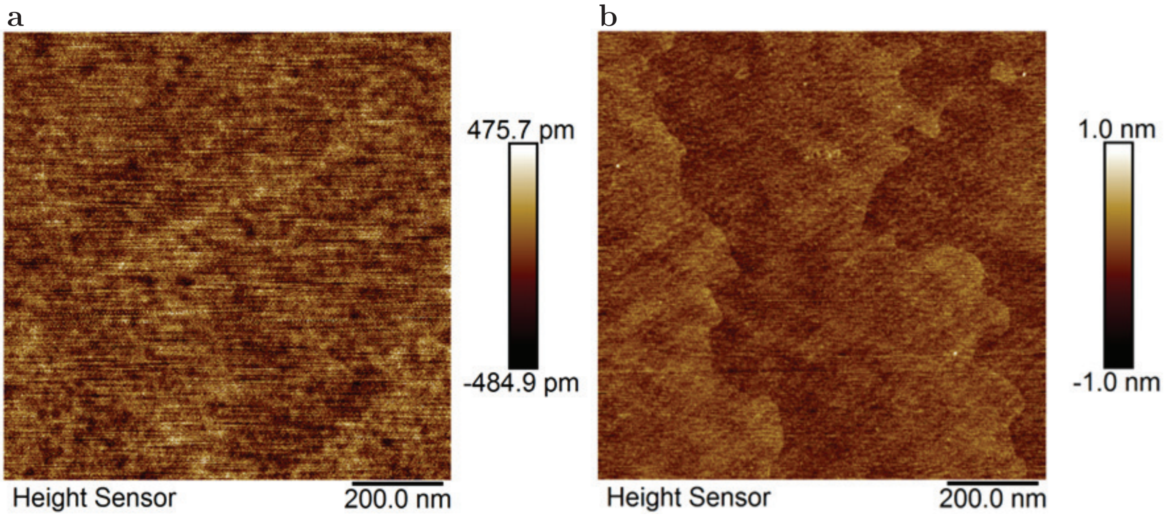

Fig. S11: **Atomic force microscopy of sapphire surface** before annealing (**a**) and after annealing (**b**).

Atomic Force Microscopy (AFM) on sapphire substrates was conducted before and after annealing using a Bruker Dimension Fastscan AFM. Surfaces before annealing had sub-nanometer roughness and uniform surface topology with no distinguishable features (Fig. S11a). After annealing, surfaces were atomically flat and displayed a terraced structure typically seen for annealed c-plane sapphire (Fig. S11b)[6, 7]. The terraces have step height of around 220 pm, approximately equal to the inter-atomic spacing in the c-axis ($c/6 = 216$ pm), and width 420 nm related to the miscut angle of the wafer, which in this case is calculated to be approximately 0.03°.

Supplementary Note 7: Tantalum film characterization

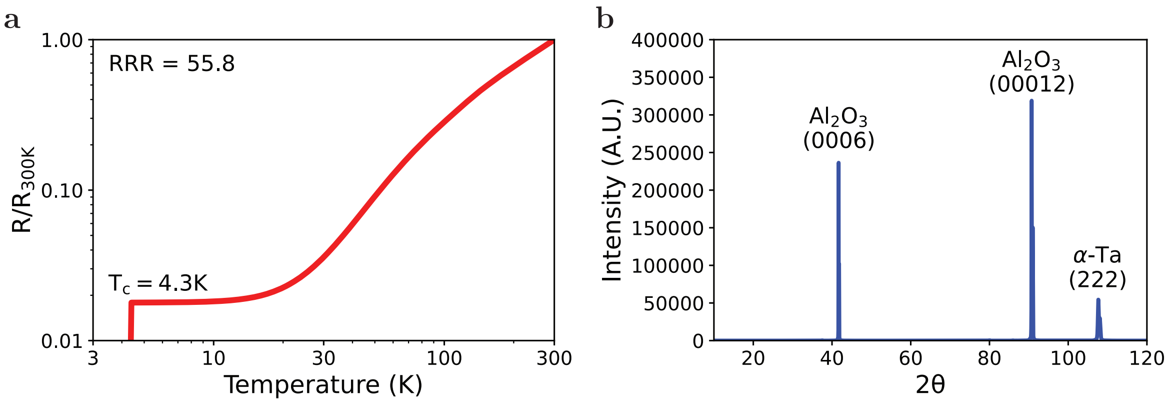

Fig. S12: **Tantalum film characterization** (**a**) DC resistance as a function of temperature. The sharp drop in resistance at $T = 4.3$ K indicates the emergence of the superconducting state. (**b**) X-ray diffractometry (XRD) of a Ta film. This particular film was entirely in the (111) orientation; other films were either entirely (110) or a mixture of (111) and (110) (not shown).

Tantalum films sputtered at 800 °C were consistently in the α phase. Resistance as a function of temperature was measured using a Quantum Design PPMS DynaCool for multiple samples, all of which had $T_c > 4.17$ K and RRR > 15, with our best sample having $T_c = 4.3$ K and RRR $= 55.8$ (Fig. S12a). All films grown this way whose crystal structure was measured using a Rigaku Miniflex II XRD confirmed the dominant presence of α-Ta growing in either the (111) or (110) orientation, while the β phase was not observed (Fig. S12b).

Supplementary Note 8: TEM film characterization

TEM of Al/AlO$_x$/Al junctions and Ta thin films was performed using an FEI Talos F200X (Fig. S13). The measured samples had a thin layer of gold sputtered on them as part of the sample preparation process. Significant differences were observed in the metal-substrate (MS) interface of the two films. While the tantalum/sapphire interface was free of amorphous material and displayed nearly epitaxial growth, the aluminum/sapphire interface has a thin ($\approx$ 2 nm) amorphous region. Additionally, the metal-air (MA) interface of tantalum has a thin ($\approx$ 3 nm) oxide layer, while the MA interface of aluminum has an $\approx$ 5 nm oxide layer. Between the two layers of aluminum lies the Josephson junction oxide, which is approximately $\approx$ 2 nm and looks amorphous, similar to the aluminum MA and MS interfaces.

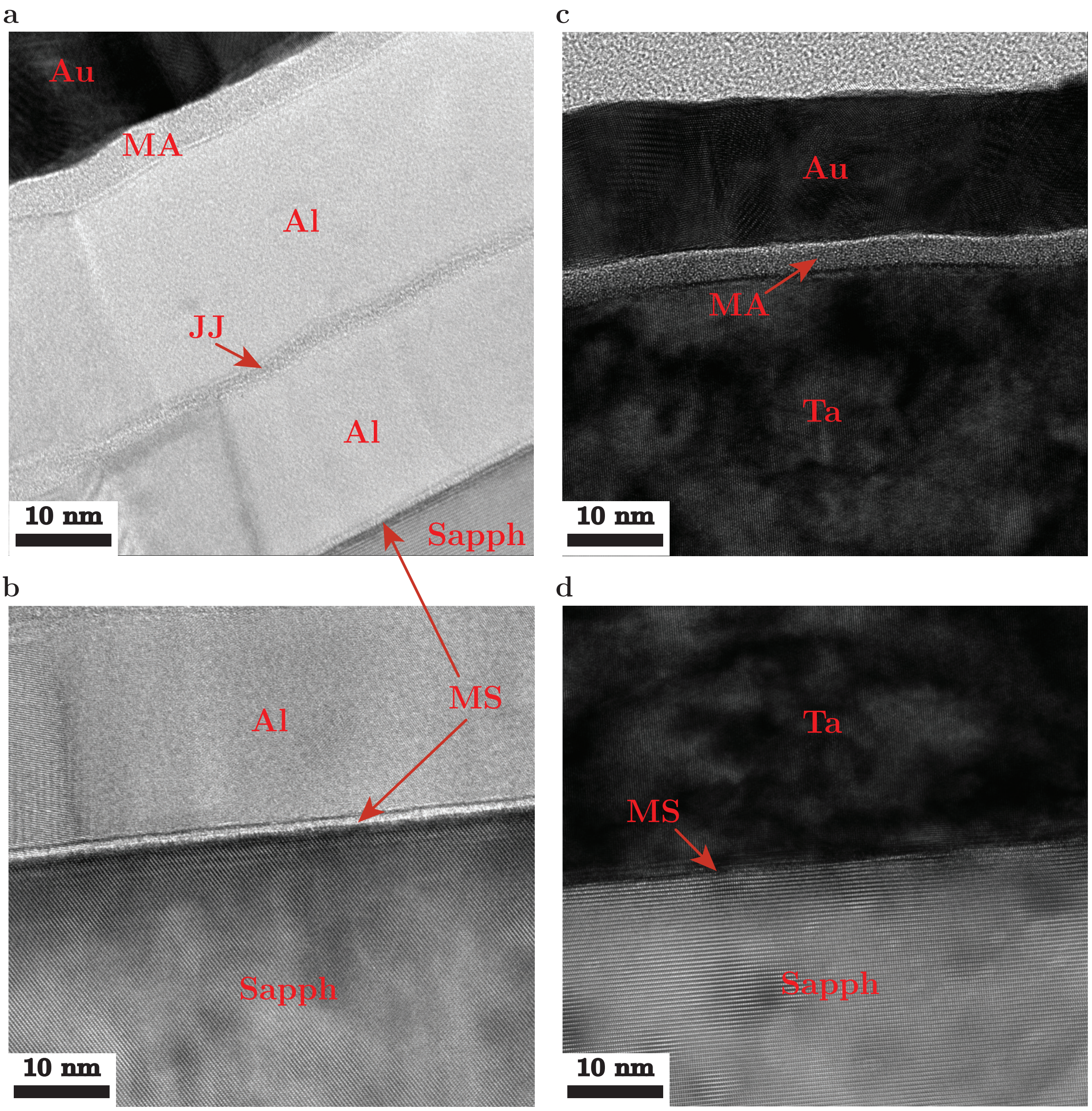

Fig. S13: **TEM of Al and Ta films. a, b** TEM of the MS and MA interfaces of a typical Al/AlO$_x$/Al film. The MA interface appears amorphous and has general stoichiometry AlO$_x$. The lower and upper Al layers are the two Josephson junction electrodes, and the AlO$_x$ between them act as the tunnel barrier. **c, d** TEM of the MS and MA interfaces of a typical Ta thin film. Like the MA interface of the Al films, the MA interface of the Ta film also appears amorphous, with general stoichiometry TaO$_x$. Gold is sputtered on top of the sample to protect the films from the TEM sample preparation process.

Supplementary Note 9: Efficient frequency-domain sampling for resonator measurements in a vector network analyzer (VNA)

The default sweep type of a standard VNA is linear, i.e. it samples frequency points uniformly across the desired frequency span. In the standard circle fitting algorithm[8], a point in frequency space translates to a point in the complex plane according to the frequency-phase relation:

$$\tan\theta = 2Q_\mathrm{L}\left(\frac{f}{f_\mathrm{r}} - 1\right). \tag{2}$$

Due to the nonlinearity of this relation, a set of S_{21} values uniformly parameterized by frequency is not uniformly parameterized by phase; the density of points is highest at the off-resonant point of the circle and lowest at the on-resonant point. Given a fixed number of points, we can improve fitting reliability by customizing the distribution of points in frequency space such that we realize a uniform distribution of points in phase space[9]. Experimentally, we employ the alternative segment sweep type provided by the Agilent E5071C VNA.

To counteract the bunching of points towards the off-resonant point, we can sample a higher density of frequency points around resonance. A simple resolution, which is adopted for many of our sweeps, is a frequency spacing that increases quadratically with each point away from the center frequency, yielding a more uniform phase distribution.

We also present the framework for a more optimal frequency distribution. Determining the N optimal frequency points is not as straightforward as taking $\theta_n = 2\pi n/N$, however. Firstly, traversing the full circumference of the circle requires sweeping from $f = -\infty$ to $f = +\infty$, which is of course unphysical; secondly, we have little knowledge of Q_L prior to measurement and circle fitting. To this end, we devise a formula built around one parameter, the frequency span of measurement expressed in terms of the number of linewidths. In practice, we qualitatively estimate the linewidth before fitting by calculating the approximate full width at half maximum (FWHM).

Suppose that we will eventually sweep a phase span $\Delta\theta \in [0, \pi]$, which is related to the frequency span Δf by:

$$\tan\Delta\theta = \frac{2\frac{Q_\mathrm{L}\Delta f}{f_\mathrm{r}}}{1 - \left(\frac{Q_\mathrm{L}\Delta f}{f_\mathrm{r}}\right)^2}. \tag{3}$$

Given that the center frequency is set to be equal to the approximate resonant frequency. By defining the linewidth as $\kappa = f_\mathrm{r}/Q_\mathrm{L}$ and introducing a parameter that we call the weight $W = \Delta f/\kappa$, we can rewrite Eq. (3) in terms of only the weight:

$$\tan\Delta\theta = \frac{2W}{1 - W^2}. \tag{4}$$

We note that the weight is the aforementioned frequency-span-to-linewidth ratio, and is named so because it ultimately determines the weighting of the point distribution towards the on-resonant point of the circle. We find that aiming for $W \approx 5$ sweeps enough of the circle for a reliable fitting.

Using the frequency-phase relation to express the nth frequency point f_n:

$$f_n = f_\mathrm{r} + \frac{f_\mathrm{r}}{2Q_\mathrm{L}}\tan\left(\frac{n}{N}\Delta\theta\right) = f_\mathrm{r} + \frac{\Delta f}{2W}\tan\left(\frac{n}{N-1}\tan^{-1}\frac{2W}{1-W^2}\right). \tag{5}$$

The points are indexed such that for an odd number of points N, n runs from $-(N-1)/2$ to $(N-1)/2$. Eq. (5) contains three parameters that can be fixed prior to fitting: the center frequency, the frequency span and the weight. Because the weight is likely to be an qualitative estimate, the frequency points f_n need to be rescaled such that the highest frequency point is $f_{(N-1)/2} = f_\mathrm{r} + \Delta f/2$, and similarly for the lowest frequency point. We introduce the ratio of the desired frequency span to the erroneous span as a result of W being an estimate:

$$R = \frac{\Delta f/2}{\frac{\Delta f}{2W}\tan\left(\frac{1}{2}\tan^{-1}\frac{2W}{1-W^2}\right)} = \frac{W}{\tan\left(\frac{1}{2}\tan^{-1}\frac{2W}{1-W^2}\right)}, \tag{6}$$

which modifies Eq. (6) to become:

$$f_n = f_\mathrm{r} + R\frac{\Delta f}{2W}\tan\left(\frac{n}{N-1}\tan^{-1}\frac{2W}{1-W^2}\right). \quad (7)$$

This rescaling offers the flexibility of adjusting the nonlinearity of the frequency distribution through W. The larger the weight, the higher the density of points at the on-resonant point; the limit of $W \to 0$ recovers the linear sweep. We find that the number of points required for a reliable fitting can be reduced by a factor of ≈ 5 by using an appropriate segment sweep, thereby shortening measurement duration by the same factor.

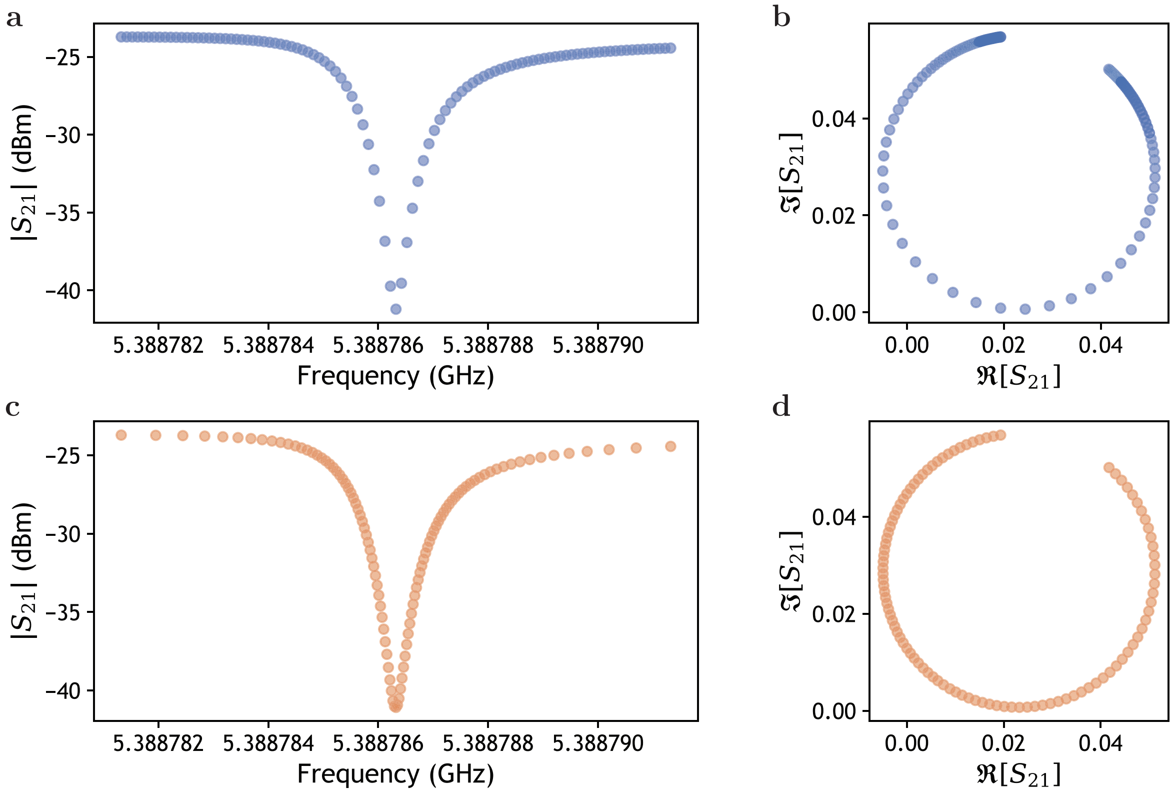

Fig. S14: $S_{21}(f)$ **amplitude and complex response with different sampling. a, b** Linear frequency sweep with $N = 101$ points, where sampled points are equally spaced in frequency. **c, d** A $W = 5$ segment sweep using $N = 101$ points. The sampled points are now more densely packed about the resonance point and equally spaced in the complex plane. Data is taken at high power from the D2 mode of an aluminum tripole stripline device fabricated on annealed HEM sapphire.

Supplementary Note 10: Derivation of resonator average photon number

We derive the average photon number in a resonator in hanger configuration using input-output theory. For a system characterized by stationary mode operator $\hat{a}$ coupled to an environment characterized by propagating mode operators $\hat{a}_{\text{in}}$ and $\hat{a}_{\text{out}}$ with energy loss rate γ, the time evolution of $\hat{a}$ is determined by the quantum Langevin equation:

$$\frac{d}{dt}\hat{a}(t) = \frac{i}{\hbar}[\hat{H}, \hat{a}(t)] - \frac{\gamma}{2}\hat{a}(t) + \sqrt{\gamma}\,\hat{a}_{\text{in}}(t)\,, \tag{8}$$

where $\hat{H}$ is the Hamiltonian of the system. The three terms on the right-hand side denote conservative time evolution, dissipation and drive respectively, and without dissipation and drive we recover the Heisenberg equation of motion. To apply input-output theory to a resonator in the hanger configuration, we introduce an internal loss channel with energy loss rate γ_{int}, and two coupling channels that describe coupling to right- and left-propagating waves with energy loss rates γ_+ and γ_- respectively. The rates are related to their respective Q-factors by $Q_{\text{int}} = \omega_{\text{r}}/\gamma_{\text{int}}$, $Q_+ = \omega_{\text{r}}/\gamma_+$ and $Q_- = \omega_{\text{r}}/\gamma_-$.

The quantum Langevin equation has one dissipation term and one drive term for each of the three channels:

$$\frac{d}{dt}\hat{a}(t) = \frac{i}{\hbar}[\hat{H}, \hat{a}(t)] - \frac{\gamma_{\text{L}}}{2}\hat{a}(t) + \sqrt{\gamma_{\text{int}}}\,\hat{a}_{\text{in,int}}(t) + \sqrt{\gamma_+}\,\hat{a}_{\text{in},+}(t) + \sqrt{\gamma_-}\,\hat{a}_{\text{in},-}(t), \tag{9}$$

where we have combined the three dissipation terms into one by defining the total (loaded) energy loss rate $\gamma_{\text{L}} = \gamma_{\text{int}} + \gamma_+ + \gamma_-$. Because we have imposed the existence of three channels, there are three boundary conditions, obtained by matching the amplitudes of the mode operators between the system and each of the three channels:

$$\begin{aligned} \hat{a}_{\text{in,int}}(t) - \hat{a}_{\text{out,int}}(t) &= \sqrt{\gamma_{\text{int}}}\,\hat{a}(t) \\ \hat{a}_{\text{in},+}(t) - \hat{a}_{\text{out},+}(t) &= \sqrt{\gamma_+}\,\hat{a}(t) \\ \hat{a}_{\text{in},-}(t) - \hat{a}_{\text{out},-}(t) &= \sqrt{\gamma_-}\,\hat{a}(t)\,. \end{aligned} \tag{10}$$

Since we drive only via the right-propagating coupling channel, Eq. (9) reduces to:

$$\frac{d}{dt}\hat{a}(t) = \frac{i}{\hbar}[\hat{H}, \hat{a}(t)] - \frac{\gamma_{\text{L}}}{2}\hat{a}(t) + \sqrt{\gamma_+}\hat{a}_{\text{in},+}(t)\,. \tag{11}$$

For a harmonic oscillator, the frequency domain expression is:

$$-i\omega\hat{a}(\omega) = -i\omega_{\text{r}}\hat{a}(\omega) - \frac{\gamma_{\text{L}}}{2}\hat{a}(\omega) + \sqrt{\gamma_+}\hat{a}_{\text{in},+}(\omega) \implies \hat{a} = \frac{\sqrt{\gamma_+}}{\frac{\gamma_{\text{L}}}{2} - i(\omega - \omega_{\text{r}})}\hat{a}_{\text{in},+}, \tag{12}$$

where the explicit dependence of the operators on ω has been omitted for clarity. From Eq. (12), the number operator is:

$$\hat{n} = \hat{a}^\dagger\hat{a} = \frac{\gamma_+}{\frac{\gamma_{\text{L}}^2}{4} + (\omega - \omega_{\text{r}})^2}\hat{a}^\dagger_{\text{in},+}\hat{a}_{\text{in},+}. \tag{13}$$

For energy to be conserved, the input power must be equal to the power dissipated. We assume that we drive at a single frequency ω:

$$P_{\text{in}} = \hbar\omega\,\langle\hat{a}^\dagger_{\text{in},+}\hat{a}_{\text{in},+}\rangle\,. \tag{14}$$

Using Eqs. (13)-(14), the average photon number $\overline{n} = \langle\hat{n}\rangle$ is:

$$\overline{n} = \frac{\gamma_+}{\frac{\gamma_{\text{L}}^2}{4} + (\omega - \omega_{\text{r}})^2}\frac{P_{\text{in}}}{\hbar\omega}. \tag{15}$$

Rewriting the rates in terms of their respective quality factors:

$$\overline{n} = \frac{\frac{4Q_{\mathrm{L}}^2}{\omega_r Q_+}}{1 + 4Q_{\mathrm{L}}^2(\frac{\omega}{\omega_r} - 1)^2} \frac{P_{\mathrm{in}}}{\hbar\omega}. \tag{16}$$

If the drive is on-resonance $\omega = \omega_r$ and the coupling is symmetric $Q_+ = 2Q_{\mathrm{c}}$:

$$\overline{n} = \frac{2}{\hbar\omega_{\mathrm{r}}^2} \frac{Q_{\mathrm{L}}^2}{Q_{\mathrm{c}}} P_{\mathrm{in}}. \tag{17}$$

The input power P_{in} is defined as the power applied to the device under test, and not the output power of the VNA. Precise determination of P_{in} necessitates proper characterization of the total attenuation of input line that extends from the VNA to the device in the dilution refrigerator. The input line (Fig. S1) consists of a number of discrete attenuators and several feet of SMA cabling whose frequency-dependent line attenuation must also be characterized. We carefully measure the input line attenuation as a function of frequency including the discrete attenuators at room temperature by measuring the attenuation using a VNA. This line attenuation is then applied to determine P_{in} at the device. Because the majority of the input line cabling uses stainless steel conductors whose attenuation changes very little with temperature, we estimate that the deviation in line attenuation when cooled to cryogenic temperatures is small; we estimate a ± 1 dB uncertainty in the determination of P_{in}.

Supplementary Note 11: Estimating systematic error from the weighted surface loss factor

The weighted surface loss factor $\Gamma_{\mathrm{surf}} = \sum_{k=\mathrm{SA,MS,MA}} \frac{p_k}{p_{\mathrm{surf}}} \frac{t_{k0}}{t_{\mathrm{surf}}} \frac{\epsilon_{\mathrm{r}0}}{\epsilon_{\mathrm{r}}} \tan\delta_k$ as defined in the Methods section "Calculation of participation ratios" is geometry-dependent due to the presence of the participation ratio terms p_k/p_{surf} that weight the loss tangents of the SA, MS, and MA regions. These terms are roughly the same for devices measured in this work and render Γ_{surf} effectively geometry-independent. However, small variations in these terms can lead to systematic errors in in the extraction of Γ_{surf} and the predictions of surface losses in transmons and hairpin stripline quantum memories. Fortunately, variations in p_k/p_{surf} are relatively small (Table S12), and result in systematic variations of 4-11% in the effective Γ_{surf} for each device. Since some devices have very low surface participation, the error propagated on the predicted quality factor is decreased further. Furthermore, the device-to-device variation that defines the uncertainties of the loss factors in Table S3 is far larger than this systematic error; therefore, the systematic error is a small effect.

Table S12: Surface loss factor weights

Device	$p_{\mathrm{SA}}/p_{\mathrm{surf}}$	$p_{\mathrm{MS}}/p_{\mathrm{surf}}$	$p_{\mathrm{MA}}/p_{\mathrm{surf}}$
Tripole D1	0.52	0.44	0.04
Tripole D2	0.54	0.43	0.03
Tripole C	0.55	0.41	0.04
Transmon	0.53	0.42	0.05
Hairpin Stripline	0.58	0.39	0.03

Table S13: TLS fit parameters

Device ID	D1 Mode				D2 Mode				C Mode			
	Q_0	$\tan\delta_{\mathrm{TLS}}$	n_c	β	Q_0	$\tan\delta_{\mathrm{TLS}}$	n_c	β	Q_0	$\tan\delta_{\mathrm{TLS}}$	n_c	β
AM22 TSL1	7.1×10^{6}	2.9×10^{-3}	0.1	0.50	4.6×10^{7}	3.1×10^{-3}	28	0.22	2.1×10^{7}	4.1×10^{-3}	1.1	0.22
AM22 TSL2	4.2×10^{6}	3.6×10^{-3}	0.1	0.47	3.3×10^{7}	2.7×10^{-3}	140	0.26	6.2×10^{6}	3.7×10^{-3}	12	0.44
AM22 TSL3	2.2×10^{6}	8.2×10^{-2}	1.0×10^{-8}	0.42	1.8×10^{7}	2.7×10^{-3}	200	0.27	8.7×10^{6}	1.9×10^{-3}	4700	0.64
DZ22 TSL3	2.5×10^{7}	2.2×10^{-3}	0.6	0.59	2.0×10^{7}	2.4×10^{-3}	0.7	0.29	3.8×10^{6}	2.9×10^{-3}	6.0	0.55
DZ22 TSL4	4.2×10^{7}	1.1×10^{-3}	5.6	0.88	7.1×10^{6}	1.3×10^{-3}	120	0.61	9.4×10^{6}	2.1×10^{-3}	45	0.30
A23Al TSL2	1.0×10^{8}	7.9×10^{-4}	6.8	0.81	2.0×10^{8}	1.4×10^{-3}	1.2	0.44	1.3×10^{8}	2.0×10^{-3}	0.9	0.35
A23Al TSL3	7.0×10^{7}	1.8×10^{-3}	0.8	0.76	7.8×10^{7}	1.9×10^{-3}	1.0	0.52	3.0×10^{7}	1.8×10^{-3}	1.0	0.48
EF21 TSL1	4.0×10^{7}	3.7×10^{-4}	9.0	0.55	1.8×10^{14}	4.7×10^{-3}	2.7×10^{6}	0.65	2.5×10^{7}	2.8×10^{-3}	5.6×10^{4}	0.70
EF21 TSL2	2.8×10^{7}	4.9×10^{-4}	4.2	0.53	2.4×10^{13}	4.7×10^{-3}	4.4×10^{5}	0.59	1.0×10^{11}	4.8×10^{-3}	8.1×10	0.39
EF21 TSL3	5.2×10^{7}	6.3×10^{-4}	4.7	0.47	3.9×10^{7}	7.3×10^{-3}	1.9×10^{6}	0.71	8.5×10^{5}	2.9×10^{-3}	6.0×10^{4}	0.55
EF21 TSL4[1]	2.5×10^{7}	8.1×10^{-4}	0.3	0.48	2.1×10^{13}	8.1×10^{-3}	6.3×10^{5}	0.62	4.8×10^{5}	-	-	-
EC21 ASL1[2]	2.0×10^{7}	7.6×10^{-4}	0.5	0.56	-	-	-	-	5.5×10^{6}	5.6×10^{-4}	430	0.76
EC21 ASL2[2]	1.4×10^{7}	3.6×10^{-4}	26	0.60	-	-	-	-	1.3×10^{7}	4.5×10^{-4}	280	0.61
EC21 ASL3[2]	1.9×10^{7}	4.2×10^{-4}	33	0.53	-	-	-	-	1.2×10^{7}	6.9×10^{-4}	38	0.63
EC21 ASL4[2]	1.2×10^{7}	6.8×10^{-4}	3.0	0.74	-	-	-	-	2.3×10^{7}	8.8×10^{-4}	48	0.34
R22 TSL1	1.5×10^{7}	6.8×10^{-4}	0.8	0.72	1.1×10^{8}	1.2×10^{-3}	4.7	0.29	1.0×10^{8}	1.8×10^{-3}	78	0.15
R22 TSL3	1.8×10^{7}	3.8×10^{-4}	13	0.83	5.8×10^{7}	6.4×10^{-4}	400	0.47	9.0×10^{6}	1.8×10^{-3}	380	0.12
R22 TSL4	1.0×10^{8}	6.3×10^{-4}	1.4	0.50	8.0×10^{7}	1.7×10^{-3}	0.2	0.21	1.7×10^{7}	6.5×10^{-4}	6600	0.43
BF22 TSL1	2.7×10^{6}	1.1×10^{-3}	1.1×10^{-3}	0.32	3.1×10^{7}	5.8×10^{-4}	200	0.37	2.0×10^{7}	1.4×10^{-3}	0.2	0.17
BF22 TSL2	1.0×10^{8}	4.0×10^{-4}	4.5	0.43	6.0×10^{7}	6.1×10^{-4}	280	0.33	2.1×10^{7}	1.2×10^{-3}	0.8	0.21
BF22 TSL3	9.5×10^{13}	3.7×10^{-4}	160	0.46	7.4×10^{7}	3.1×10^{-4}	1700	0.60	1.2×10^{7}	5.2×10^{-4}	8800	0.49
BF22 TSL4	1.6×10^{8}	4.3×10^{-4}	2.8	0.44	4.9×10^{7}	4.0×10^{-4}	2600	0.50	2.5×10^{7}	1.3×10^{-3}	1.7	0.15

[1] EF21 TSL4 C mode was found to be power independent and the best interpolating function was found to be a constant value of Q_0.

[2] ASL has only a differential (D) mode and a common (C mode)

TSL: Tripole stripline
ASL: Adjacent stripline

References

[1] Lei, C.U., Ganjam, S., Krayzman, L., Banerjee, A., Kisslinger, K., Hwang, S., Frunzio, L., Schoelkopf, R.J.: Characterization of microwave loss using multimode superconducting resonators. Physical Review Applied **20**, 024045 (2023)

[2] Lei, C.U., Krayzman, L., Ganjam, S., Frunzio, L., Schoelkopf, R.J.: High coherence superconducting microwave cavities with indium bump bonding. Appl. Phys. Lett. **116**, 154002 (2020)

[3] Brecht, T., Reagor, M., Chu, Y., Pfaff, W., Wang, C., Frunzio, L., Devoret, M.H., Schoelkopf, R.J.: Demonstration of superconducting micromachined cavities. Applied Physics Letters **107**(19), 192603 (2015)

[4] Paik, H., Schuster, D.I., Bishop, L.S., Kirchmair, G., Catelani, G., Sears, A.P., Johnson, B.R., Reagor, M.J., Frunzio, L., Glazman, L.I., Girvin, S.M., Devoret, M.H., Schoelkopf, R.J.: Observation of high coherence in josephson junction qubits measured in a three-dimensional circuit QED architecture. Physical Review Letters **107**, 240501 (2011)

[5] Yan, F., Gustavsson, S., Kamal, A., Birenbaum, J., Sears, A.P., Hover, D., Gudmundsen, T.J., Rosenberg, D., Samach, G., Weber, S., Yoder, J.L., Orlando, T.P., Clarke, J., Kerman, A.J., Oliver, W.D.: The flux qubit revisited to enhance coherence and reproducibility. Nature Communications **7**(12964) (2016)

[6] Kamal, A., Yoder, J.L., Yan, F., Gudmundsen, T.J., Hover, D., Sears, A.P., Welander, P., Orlando, T.P., Gustavsson, S., Oliver, W.D.: Improved superconducting qubit coherence with high-temperature substrate annealing. arXiv preprint arXiv:1606.09262 (2016)

[7] Crowley, K.D., McLellan, R.A., Dutta, A., Shumiya, N., Place, A.P.M., Le, X.H., Gang, Y., Madhavan, T., Bland, M.P., Chang, R., Khedkar, N., Feng, Y.C., Umbarkar, E.A., Gui, X., Rodgers, L.V.H., Jia, Y., Feldman, M.M., Lyon, S.A., Liu, M., Cava, R.J., Houck, A.A., Leon, N.P.: Disentangling losses in tantalum superconducting circuits. Physical Review X **13**, 041005 (2023)

[8] Probst, S., Song, F.B., Bushev, P.A., Ustinov, A.V., Weides, M.: Efficient and robust analysis of complex scattering data under noise in microwave resonators. Review of Scientific Instruments **86**(2), 024706 (2015)

[9] Baity, P.G., Maclean, C., Seferai, V., Bronstein, J., Shu, Y., Hemakumara, T., Weides, M.: Circle fit optimization for resonator quality factor measurements: point redistribution for maximal accuracy. arXiv preprint arXiv:2301.06364 (2023)
